# ‘Doing’ or ‘using’ intersectionality? Opportunities and challenges in incorporating intersectionality into knowledge translation theory and practice

**DOI:** 10.1186/s12939-021-01509-z

**Published:** 2021-08-21

**Authors:** Christine Kelly, Danielle Kasperavicius, Diane Duncan, Cole Etherington, Lora Giangregorio, Justin Presseau, Kathryn M. Sibley, Sharon Straus

**Affiliations:** 1 Department of Community Health Sciences, University of Manitoba, Room S108-E - 750 Bannatyne Avenue, Winnipeg, MB R3E 0W3 Canada; 2 Li Ka Shing Knowledge Institute, St. Michael’s Hospital, Toronto, Canada; 3 Accelerating Change Transformation Team (ACTT), Alberta Medical Association, Edmonton, Alberta Canada; 4 Clinical Epidemiology Program, Ottawa Hospital Research Institute, Ottawa, Canada; 5 Department of Kinesiology and Schlegel-UW Research Institute for Aging, University of Waterloo, Waterloo, Canada; 6 KITE-Toronto Rehabilitation Institute, University Health Network, Toronto, Canada; 7 School of Epidemiology and Public Health, University of Ottawa, Ottawa, Canada; 8 George and Fay Yee Centre for Healthcare Innovation, Winnipeg, Manitoba Canada; 9 Department of Medicine, University of Toronto, Toronto, Canada

**Keywords:** Intersectionality, Feminist theory, Health equity, Interdisciplinary research

## Abstract

Intersectionality is a widely adopted theoretical orientation in the field of women and gender studies. Intersectionality comes from the work of black feminist scholars and activists. Intersectionality argues identities such as gender, race, sexuality, and other markers of difference intersect and reflect large social structures of oppression and privilege, such as sexism, racism, and heteronormativity. The reach of intersectionality now extends to the fields of public health and knowledge translation. Knowledge translation (KT) is a field of study and practice that aims to synthesize and evaluate research into an evidence base and move that evidence into health care practice. There have been increasing calls to bring gender and other social issues into the field of KT. Yet, as scholars outline, there are few guidelines for incorporating the principles of intersectionality into empirical research. An interdisciplinary, team-based, national health research project in Canada aimed to bring an intersectional lens to the field of knowledge translation. This paper reports on key moments and resulting tensions we experienced through the project, which reflect debates in intersectionality: discomfort with social justice, disciplinary divides, and tokenism. We consider how our project advances intersectionality practice and suggests recommendations for using intersectionality in health research contexts. We argue that while we encountered many challenges, our process and the resulting co-created tools can serve as a valuable starting point and example of how intersectionality can transform fields and practices.

## Introduction

Intersectionality is a widely adopted theoretical orientation in women and gender studies. Growing from the work of black feminist scholars and activists, intersectionality argues that individual identities such as gender, race, sexuality, and others, overlap and intersect and reflect macro-level forms of oppression and privilege, such as sexism, racism, and heteronormativity [11]. Intersectionality transformed the field of women’s studies, which previously focused exclusively on gender. It is so well established in women and gender studies that it has been described as a ‘fast travelling concept’ [26] that risks becoming a ‘buzzword’ [13]. Outside of the humanities and social sciences, there are efforts to integrate intersectionality into health-related disciplines, with the majority of papers published around 2010 onward [29]. Health researchers and theorists agree - there is limited guidance for incorporating this relevant, evolving concept into empirical research [7, 34].

This commentary presents a thematic literature review related to intersectionality based in the tradition of the social sciences in order to provide general descriptive context [16]. We then reflect on a specific example from an interdisciplinary, team-based, national research project in Canada that aimed to bring an intersectional lens to the field of knowledge translation. Knowledge translation is dynamic and iterative process that includes the synthesis, dissemination, exchange and ethically sound application of knowledge to improve health […], provide more effective health services and products, and strengthen the health care system’ (Graham, 2010, cited in [4]).

We argue the radical history of intersectionality has the potential to enrich mainstream health research, yet remains challenging to incorporate within the confines of existing research norms.

## Evolutions and recent literature related to intersectionality

Intersectionality comes from black feminist writers and activists who were excluded from mainstream feminist and anti-racism movements [10, 11]. Intersectionality represents an implicit critique of exclusion and erasure of difference. Intersectionality argues that oppression and privilege can shift depending on the context, and that all experiences of marginalization are relevant [5]. In the context of public health, Bowleg [2] outlines three tenets of intersectionality: first, that social identities are not independent ‘but multiple and intersecting;’ second, people from historically oppressed and marginalized groups are the focal point; and finally, intersectionality can help reveal disparate health outcomes. Hankvisky made major contributions to bring intersectionality to health research, arguing that a focus on difference will advance women’s health research [18–21].

Despite the long history in women and gender studies, and the work of Bowleg, Hankvisky, and others, intersectionality is still not mainstream in health research. Many health disciplines regard theoretical research and even qualitative work as not trustworthy or rigorous, making it challenging to widely incorporate intersectionality [8]. One approach is to convert intersectionality into a quantitative measure [1, 14, 28, 29, 35, 36]. For example, Scheim and Bauer [36] developed the Intersectionality Discrimination Index (InDI). In a commentary on Scheim and Bauer’s measure, Harnois and Bastos [22] are generally supportive, although point out ‘its inability to distinguish among intersectional, multiple, and single-axes experiences of discrimination.’ Nevertheless, these measures demonstrate a growing interest in incorporating intersectionality into health-related fields.

Meanwhile scholars in women and gender studies (and related fields such as sociology, social work, and political science) continue to theorize the high-level concept of intersectionality (e.g., [5, 34]). Recent theorizing on intersectionality is explicitly concerned with social justice, unlike the work in health that focuses only on social identities. For example, Rice et al. [34] trace a genealogy of intersectionality ‘to identify challenges in translating the concept into research methods.’ They define three ‘critical movements’ of the concept—that is, key points of contestation. First, they explore its aims, contrasting how intersectionality can be used to merely ‘manage complexity’ versus understand oppression; then its scope, considering whether intersectionality should only explore black women’s experiences versus ever-emerging social identities; and finally axioms, that is, whether intersectionality theorizes subjectivities as static rather than relational (such as in queer theory).

Health-related applications of intersectionality do not always reflect the ongoing theorizing or the overtly political nature of intersectionality found in women and gender studies. Public health scholars Green, Evans and Subramanian [17] observe this disconnect and warn:

Intersectionality will be most effective where combined with social theory on the production of health inequalities. To do otherwise is to lose the focus intersectionality theory brings to underlying power structures and social determinants, and to treat the identities themselves as being inherently ‘risky’ rather than as proxies for social position and experience. (p. 215)

Or more simply, ‘Attention to process, rather than simply outcomes, is critical’ ([22], p. 75). In an epistemological sense, the literal and imagined hierarchy of evidence in health research often excludes theorizing from women and gender studies from major journals, ironically presumed to be somewhat ‘irrelevant’ to the practice of intersectionality in health. A truly interdisciplinary approach is essential for bringing this concept outside of the social sciences.

Our research team was largely unfamiliar with intersectionality at the outset (with the exception of the few gender/intersectionality scholars engaged in this work). Our project demonstrates some of the implications of taking up intersectionality across disciplines. Intersectionality foregrounds lived experiences and presumes that individuals may have a vastly different experience of a health condition, service, or intervention mediated by their specific identities. Our project goes beyond attempting to measure intersectionality to use it as a complete methodology-- a comprehensive approach that encompasses the research questions, analysis, dissemination, and even a re-shaping of the research process and team. We now describe a case study in attempting to operationalize intersectionality in health research.

## Case study: project description

This commentary is part of our commitment to honor intersectional reflexivity—that is, an uncomfortable and sustained evaluation of research practices and how our own identities may influence the research [33]. In 2017, the Canadian Institutes of Health Research launched an opportunity for team grants in gender and knowledge translation (KT). In short, KT is the science and practice of changing behaviors to move the findings of health research into health care settings. In response to this call, a group of KT researchers, citizen partners, and scholars trained in women and gender studies in Canada came together. The team originally planned to focus on sex and gender in response to the research call. As women and gender studies scholars joined the team, they advocated for an intersectional approach. In the end, we were funded for a three-year team grant aimed at helping KT developers use an intersectional approach when designing and implementing interventions to address the needs of older adults (For a complete project description, see the registered protocol and project website: [27, 39]).

We present three tensions that arose in the course of our work together and conclude with possible pathways forward to encourage more researchers to bring intersectionality to the field of KT.

## Three tensions in practice

### Discomfort with social justice

The target audience of our project is KT practitioners working to improve the health of older adults. Informed by the experiences of practitioners on the project team, we presumed that intersectional concepts would be novel and searched for an established, accessible intersectionality framework. The group selected the Intersectionality-Based Policy Analysis Framework [20]. The team chose to focus on ‘intersecting categories’ and that other principles, specifically social justice, would not be incorporated. This ‘setting aside’ of social justice during the project development phase is an important tension to note. A set of social categories (e.g., race) was identified, drawing on a model that considers the needs of disadvantaged groups in public health program implementation [31]. At a critical juncture, we decided to exclude Indigeneity from our approach, to be further discussed below.

In December 2017, we recruited additional intersectional scholars for a prioritization activity. Women and gender studies scholars quickly questioned the lack of focus on social justice in the project, and it was difficult to recruit scholars to participate in the project. The role of social justice within intersectionality frameworks is somewhat contested, although overall most intersectionality scholars will directly discuss social justice. Conversely, Collins and Bilge [9] argue ‘working for social justice is not a requirement for intersectionality’ (p.30). They suggest intersectionality is best conceptualized as analytical tool that with or without an explicit focus on social justice will achieve the same ends. Similarly, Cho, Crenshaw and McCall [6] call us to think of intersectionality as an ‘analytical sensibility’ in order to emphasize ‘what intersectionality does rather than what intersectionality is.’ On the other hand Rice et al. [34] argue that social justice is integral to using this concept: ‘Intersectionality orients to social justice, so research utilizing intersectional analysis must commit to justice in its processes and knowledge production.’

In the context of our project, we assumed that we would need to meet the KT scholars and practitioners ‘where they are at.’ We conducted barriers and facilitators interviews with KT practitioners in order to select which existing KT tool to adapt with an intersectional lens. The KT participants interpreted the social justice aims of intersectionality as “politicization” and suggested it is a barrier to using intersectionality in their work. In short, the women and gender studies scholars were comfortable with adopting a social justice framework, which is integral to the culture and theorizing of their field. Women and gender studies training and research often includes an explicit commitment to improving equity and social justice for women as well as other marginalized identities, and connections to grassroots social justice movements. The KT practitioners and scholars are trained in various health disciplines that do not have the same focus. Further, some health research methods emphasize avoiding ‘bias’ and committing to social justice may be interpreted as a form of bias by some health researchers. Feminist researchers have long challenged the ‘apolitical’ nature of research, and indeed, this is one example of the disciplinary divides we encountered in working together [33].

### Disciplinary divides

One of the more difficult aspects of incorporating intersectionality into the field of KT lies within disciplinary divides. These divides were apparent when the women and gender studies experts on the team participated in a two-day KT training course in October 2017 in order to learn the commonly used theoretical tools and concepts of the knowledge translation field.

Many KT models, theories, and frameworks do not explicitly reflect on the context that may affect individual control over behaviour change. Kitto et al. [25] suggests KT’s inevitable focus on individual behaviors ‘can result in a reductionist form of methodological individualism that de-contextualizes the behavior of the individual(s).’ Intersectionality is a lens to help KT developers use a holistic, humanistic approach, to consider that an individual or community can simultaneously experience privilege and oppression. A somewhat opposite critique applies to intersectionality – that is, it is can be too focused on big picture ideas and not enough on how to apply it.

The use of an intersectional lens is compatible with an integrated KT approach, a newer development of the KT field [24, 37]. The integrated KT approach involves end-users in the development of a project team, research question and protocols through to dissemination. Intersectionality complements the iKT approach by prompting KT practitioners to consider the lived experiences of end-users.

There is also difference in the general approaches of the fields, with KT taking a deductive application of theoretical frameworks in contrast to the inductive, grounded-theory, or grassroots approaches of intersectionality, based in lived experiences. For example, two of the frameworks we engaged with in this project can be classified as deductive. Michie et al. [30]‘s widely cited Theoretical Domains Framework outlines 14 cognitive, affective, and social barriers and facilitators to behaviour change. Secondly, the Consolidated Framework for Implementation Research (CFIR) details a ‘menu of constructs’ associated with effective implementation and is applied through a predetermined coding structure [12]. Both of these frameworks are presented as comprehensive lists of factors that affect what people do, and at the outset of the project these tools did not include factors like racism, sexism, and other broad social conditions that can influence ways people behave in specific situations.

Finally, while not unique to the field of KT, health research and evidence-based medicine privileges certain types of knowledge. The field of KT relies heavily on both the knowledge pyramid [15] and the foundational Knowledge-to-Action Cycle [15]. These tools were presented to the women and gender studies scholars during the KT training session. Classifying lived experience and qualitative scholarship as lesser forms of knowledge, or excluding them from the diagrams of what ‘counts’ as knowledge contradicts intersectionality [15, 32].

### Meaningful inclusion versus tokenism

The third tension we present is related to tokenism, which is a risk of using an intersectional approach. As part of our work, in January 2018, 37 participants engaged in facilitated in-person and online prioritization activity: 16 citizen representatives, 8 KT researchers, 9 KT practitioners, and 10 intersectionality scholars.

In our recruitment of citizen partners, we included language surrounding prioritizing ‘diverse voices,’ and our demographic polling indicates we were fairly successful in including LGBTQ+ individuals, immigrants, and older people, but less successful in including people of lower socio-economic backgrounds. In a follow-up evaluation, some participants noted that they ‘felt like there were ample opportunities to express my views and hear back from others’ In contrast, others participants noted ‘We do wonder what our role is here and whether the health team got anything truly worthwhile from our participation that you/the team hadn’t already considered.’

A second example, at the outset we outlined that the scope of this project did not extend to Indigenous communities. There were times when we would mention it or add it on to our documents, and other times when we wouldn’t. At a meeting in March 2019, it was brought up that some Indigenous scholars argue intersectionality is not an adequate framework for studying the effects of colonialism [23]. After discussion, the team confirmed that including Indigenous concepts would be beyond the scope of our work as we had not followed the OCAP (ownership, control, access, and possession) principles for conducting research with Indigenous communities, and including Indigenous people at that juncture would be tokenistic [38]. The team decided to recognize this significant limitation and consulted with an Indigenous scholar to craft a limitation statement that is featured within project outputs (see limitation statement at [27]).

One mechanism to defend against tokenism is the intersectional practice of reflexivity. Our efforts at reflexivity evolved as the project went on, and we sought REB approval from the primary health institution to collect information on the project team members’ own intersecting categories. Our team included some diversity, although not as much as we would like. Having team members who identify as women, sexual minorities, and people of color shaped our approach. The move to define our group’s intersecting categories was an important practice that we plan to continue in our work.

## Conclusion

While we encountered many challenges, our process, and the resulting tools can serve as a valuable starting point and example of how intersectionality can transform fields and practices. In Table 1, we outline our lessons learned and recommendations for others who are considering using intersectionality in health-related field to show the way forward from our tension points. In many ways our work is incomplete. There is a concern among intersectionality scholars that the concept has lost its anti-racist edge [3] and that ‘scholars may have rewritten the genealogy of intersectionality to downplay its focus on social transformation from the racialized, sexualized bottom-up to better align with dominant knowledge systems in their field’ ([34], p. 11). Reflecting on these concerns, our project did not engage with the most politicized form of intersectionality, rooted in anti-racist politics and aiming towards complete social transformation. Yet, we did ‘use’ intersectionality as an analytical framework [9] or analytical sensibility [6], in some ways resulting in us ‘doing’ it.

**Table 1:**
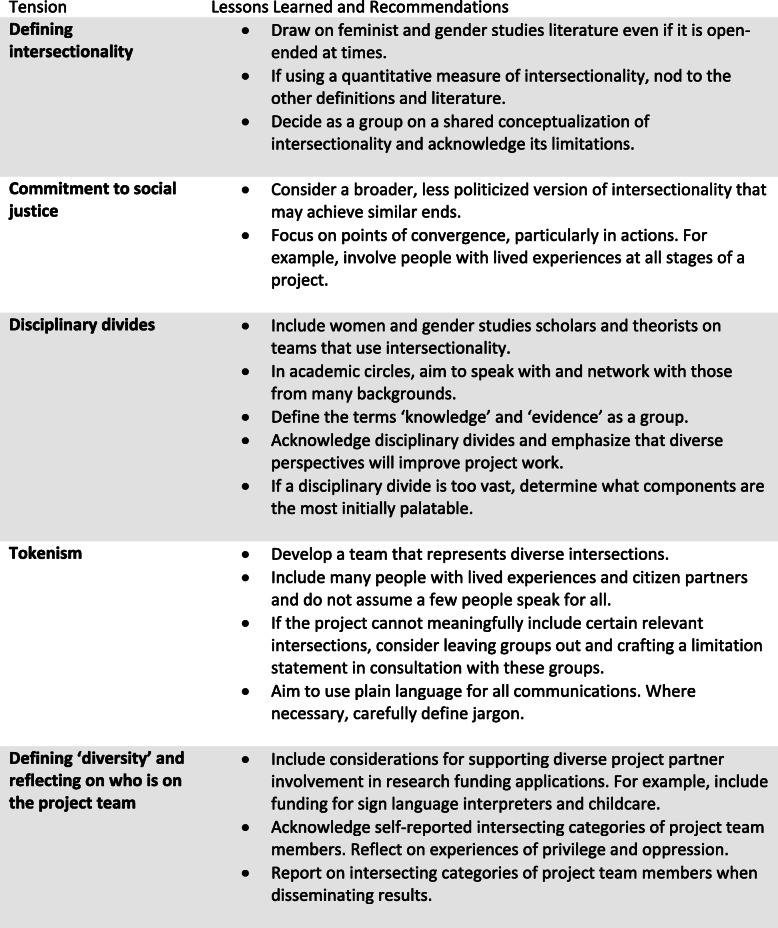
Lessons Learned

Our takeaways are to suggest we continue to work towards truly interdisciplinary projects that do not subsume the social and qualitative at the expense of empirical and quantitative. We encourage health researchers to work directly with those trained in women and gender studies, including those who are purely theoretical in their work—despite the challenges this may pose to the typical ways of doing research for both sides.

The tenets of intersectionality outlined by Bowleg [2] proved relevant to our work. Bowleg suggests people from historically oppressed groups must be the focal point to using intersectionality. It is essential to include multiple social identities in all phases of research – including in the formation of the research team, as advisory board members, and as research participants. We must work together to actively learn about intersectionality and how to apply it and demonstrate it through our practices.

Like many who have written about intersectionality, we concur that using intersectionality can be uncomfortable, but the resulting tools and our renewed commitments to intersectionality are valuable outcomes. Ultimately, this work represents a first foray into reflecting on the lived experiences of those involved in and impacted by knowledge translation research and practice.

## Data Availability

Not applicable.
